# The role of three-dimensional scaffolds based on polyglycerol sebacate/ polycaprolactone/ gelatin in the presence of Nanohydroxyapatite in promoting chondrogenic differentiation of human adipose-derived mesenchymal stem cells

**DOI:** 10.1186/s12575-023-00197-z

**Published:** 2023-03-24

**Authors:** Pardis Yousefi Talouki, Saeed Hesami Tackallou, Shahrokh Shojaei, Soheila Zamanlui Benisi, Vahabodin Goodarzi

**Affiliations:** 1 Department of Biomedical Engineering, Central Tehran Branch, Islamic Azad University, Tehran, Iran; 2 Department of Biology, Central Tehran Branch, Islamic Azad University, P.O. Box 13145-784, Tehran, Iran; 3 Stem Cell Research Center, Tissue Engineering and Regenerative Medicine Institute, Islamic Azad University, Central Tehran Branch, Tehran, Iran; 4 Applied Biotechnology Research Center, Baqiyatallah University of Medical Sciences, Tehran, Iran

**Keywords:** Biomaterials, Nanocomposites, Cartilage, Tissue engineering

## Abstract

**Background:**

Tissue engineering for cartilage regeneration has made great advances in recent years, although there are still challenges to overcome. This study aimed to evaluate the chondrogenic differentiation of human adipose-derived mesenchymal stem cells (hADSCs) on three-dimensional scaffolds based on polyglycerol sebacate (PGS) / polycaprolactone (PCL) / gelatin(Gel) in the presence of Nanohydroxyapatite (nHA).

**Materials and methods:**

In this study, a series of nHA-nanocomposite scaffolds were fabricated using 100:0:0, 60:40:0, and 60:20:20 weight ratios of PGS to PCL: Gel copolymers through salt leaching method. The morphology and porosity of prepared samples was characterized by SEM and EDX mapping analysis. Also, the dynamic contact angle and PBS adsorption tests are used to identify the effect of copolymerization and nanoparticles on scaffolds' hydrophilicity. The hydrolytic degradation properties were also analyzed. Furthermore, cell viability and proliferation as well as cell adhesion are evaluated to find out the biocompatibility. To determine the potential ability of nHA-nanocomposite scaffolds in chondrogenic differentiation, RT-PCR assay was performed to monitor the expression of collagen II, aggrecan, and Sox9 genes as markers of cartilage differentiation.

**Results:**

The nanocomposites had an elastic modulus within a range of 0.71–1.30 MPa and 0.65–0.43 MPa, in dry and wet states, respectively. The PGS/PCL sample showed a water contact angle of 72.44 ± 2.2°, while the hydrophilicity significantly improved by adding HA nanoparticles. It was found from the hydrolytic degradation study that HA incorporation can accelerate the degradation rate compared with PGS and PGS/PCL samples. Furthermore, the in vitro biocompatibility tests showed significant cell attachment, proliferation, and viability of adipose-derived mesenchymal stem cells (ADMSCs). RT-PCR also indicated a significant increase in collagen II, aggrecan and Sox9 mRNA levels.

**Conclusions:**

Our findings demonstrated that these nanocomposite scaffolds promote the differentiation of hADSCs into chondrocytes possibly by the increase in mRNA levels of collagen II, aggrecan, and Sox9 as markers of chondrogenic differentiation. In conclusion, the addition of PCL, Gelatin, and HA into PGS is a practical approach to adjust the general features of PGS to prepare a promising scaffold for cartilage tissue engineering.

**Graphical Abstract:**

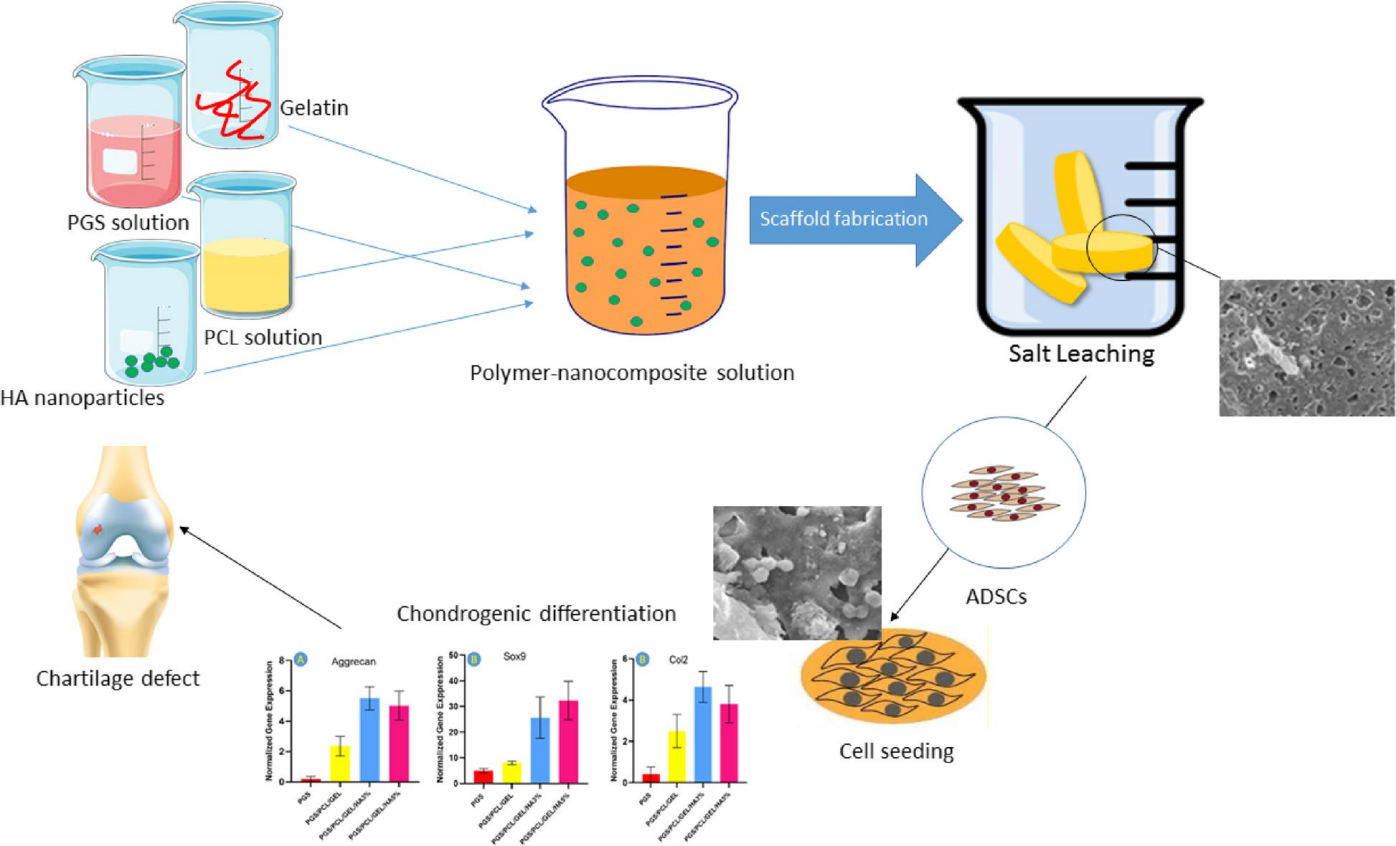

## Introduction

Cartilage, as a unique connective tissue [1], exhibits distinctive characteristics such as low intrinsic healing capacity due to its avascular nature and low cell density [2, 3]. Articular cartilage (AC) covers the surfaces of the bones in a synovial joint that plays a critical role in maintaining joint durability and mobility by providing an almost frictionless movement of the joints [4]. However, damage to the AC from wear, tears, direct trauma, and systemic diseases leads to impaired cartilage treatment which causes osteoarthritis, affecting more than 25% of the adult population globally and about 30.8 million adults in the United States [5].

Since there is no permanent clinical solution for OA treatment, cartilage tissue engineering (CTE) has been investigated with the potential to enhance the healing process. CTE conducts biodegradable materials, relevant cells like articular chondrocytes or mesenchymal stem cells (MSCs), and growth factors (GFs) to regenerate damaged or diseased tissues [5–8].

Among different types of natural and synthetic biomaterials, collagen, gelatin, fibrin, silk fibroin, alginate, hyaluronan, chondroitin sulfate, agarose, and chitosan, as well as polyethylene glycol[PEG], poly (lactide-co-glycolic) acid [PLGA], and polycaprolactone [PCL] have widely used in CTE [9–12].

In addition, recent studies have reported the incorporation of inorganic nanoparticles such as bioactive glass, tricalcium phosphate, and hydroxyapatite in natural or synthetic polymers to fabricate nanocomposite scaffolds for cartilage regeneration [13–16]. For example, Jianghong et al., demonstrated the capability of 3D-printed HA-doped scaffolds to support the proliferation and chondrogenic differentiation of human umbilical cord blood-derived mesenchymal stem cells (hUCB-MSCs), also proved that gelatin/HA films can support growth and phenotype of chondrocytes compared with gelatin alone [17]. Another studies also showed the promotion of the proliferation and migration of chondrocytes as well as chondrogenic differentiation of stem cells in hybrid materials containing HA [18–20].

Since Wang et al. [21] introduced PGS as a biodegradable synthetic polyester, many researchers have investigated PGS-based copolymers and nanomaterials to provide tissue engineering scaffolds. However, the effect of combining Gel and HA in PGS: PCL copolymer on chondrogenesis has not been evaluated so far [22, 23]. In the present study, five different composite scaffolds including PGS, PGS/PCL, PGS/PCL/Gel, PGS/PCL/Gel/HA (3% wt.), and PGS/PCL/Gel/HA(5%wt.) were synthesized for cartilage regeneration applications.

Poly(glycerol sebacate)(PGS) is a biodegradable polyester with flexible and elastomeric nature that has been extensively targeted in both soft and hard tissue engineering as well as drug delivery systems, and wound healing [24, 25]. A two-step polycondensation of glycerol and sebacic acid is used to prepare PGS, and then it can be modified using copolymers, hybrid, composite, and nanocomposite materials [13, 24]. Since PGS possesses a high degradation rate, blending of PGS and polycaprolactone (PCL) with a long deg­radation rate (1–2 years) [13] can increase the stability of PGS while improving PCL hydrophilicity, biological behavior, and cell adhesion [12]. In addition, PCL can mimic the anisotropic and viscoelastic biomechanical properties of articular cartilage [1], which makes it a promising option for cartilage tissue engineering.

Gelatin (Gel), as a natural polymer, has been widely used in tissue engineering scaffolds due to its good biocompatibility, biosafety, and chemical similarities to the extracellular matrix (ECM) in the native tissues, and the presence of functional groups that allow facile chemical modifications with other biomaterials or biomolecules [26, 27].

Despite this widespread use, poor mechanical strength and rapid decomposition limit its application. In contrast, polycaprolactone (PCL), as a biodegradable, semi-crystalline elastic polymer, has good mechanical strength and slow degradability but poor biocompatibility [26, 28–31]. Obviously, the combination of Gel and PCL is an efficient approach to achieve optimal degradation rates while improving biocompatibility [32, 33].

After preparing copolymers and chemically characterizing them, different concentrations of nano-hydroxyapatite (nHA) particles were incorporated into copolymers, and physical, microstructural, and biochemical properties of composite scaffolds were characterized concerning morphology, nano-particle distribution, wettability, bioactivity, and degradation. Finally, human adipose-derived mesenchymal stem cells were used for in vitro biological evaluations of the scaffolds to investigate cytocompatibility, cell attachment, cell proliferation, and cartilage differentiation**.**

## Materials and methods

### Materials

Glycerol, sebacic acid, Gelatin, Ɛ-caprolactone (PCL), and hydroxyapatite nanoparticles (particle size < 100 nm) were purchased from Sigma-Aldrich (USA). Chloroform and Isopropanol were ordered from Dr. Mojallali Chemical Industries Complex (Iran). Hexamethylene diisocyanate (HDI) was purchased from Merck (Germany). Phosphate buffer saline (pH 7.3) was obtained from the GIBCO. Adipose-derived mesenchymal stem cells were supplied from the Stem Cell Technology Research Center (Tehran, Iran) and used for cellular experiments.

### Methods

#### Synthesis of PGS-based copolymers and nanocomposite samples

PGS is synthesized by reacting equimolar sebacic acid and glycerol monomers through the polycondensation method. To prepare pure PGS samples, the equimolar of glycerol (G) and sebacic acid (S) monomers (G:S) (1 mol:1 mol) and also a specific amount of catalyst are stirred at 120 °C in a chemical reactor for half an hour.

**Fig. 1:**
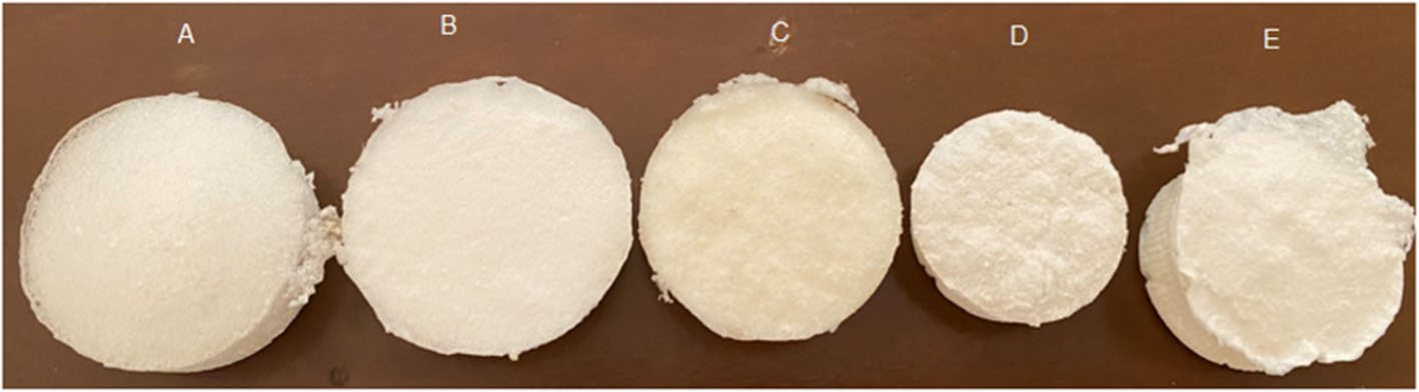
The fabricated scaffolds by salt leaching method for cell seeding, A(PGS), B(PGS/PCL), C(PGS/PCL/Gel), D(PGS/PCL/Gel/HA 3%), and E(PGS/PCL/Gel/ha 5%)

After this time, sebacic acid dissolves in glycerol and melts and pure PGS resin is obtained. Then dissolve a certain amount of PGS and PCL in DMSO at room temperature for 24 h. Synthesized pre-polymers of PGS and PGS- PCL were shown in Fig. 1. Different physical forms in this figure can show the observable influence of ε-caprolactone in the PGS structure. Synthesized polymers were totally dissolved in Chloroform and were precipitated in n-Hexane. This process was performed several times for removing unreacted monomers and residual catalysts.

To prepare PGS- PCL-Gelatin pre-polymer, dissolve gelatin in 10% acetic acid in a container and add it to the PGS- PCL polymer solution on the stirrer at room temperature for 24 h.

Furthermore, nanocomposite samples are also synthesized according to the above method. First, a specific amount of hydroxyapatite nanoparticles (3 and 5 w.t%) is dissolved in DMSO by using an ultrasonic bath (15 min), and then desired weight ratios of copolymers were added to the mixture and homogenized using a Hielscher (UP100H) homogenizer to prepare scaffolds containing 3 and 5% (w/w) of HA nanoparticles (all of volume amount of solvents was 10 cc).

#### Scaffold fabrication method

Different weight ratios of synthesized copolymers to PGS (PGS: PCL: Gel) were set at 100:0:0, 60:40:0, and 60:20:20, and dissolved in chloroform/ HDI (4:1 v/v) solvent at 10% (w/v) polymer concentration, overnight. The summary of these desired concentrations and their abbreviations were shown in Table 1.

**Table 1:** Summarized desired concentrations of the polymers and their abbreviations

Sample	PGS	PCL	Gelatin	HA
**PGS**	100	0	0	0
**PGS/PCL**	60	40	0	0
**PGS/PCL/Gel**	60	20	20	0
**PGS/PCL/Gel/HA**	60	20	20	3
**PGS/PCL/Gel/HA**	60	20	20	5

To prepare nanocomposite blends, 3 and 5% w.t of HA nanoparticles were added to PGS: PCL: Gel copolymers. To prepare porous polymeric and nanocomposite scaffolds, catalyst 1,4 Tin octanoate (10 ppm) was added to each of the polymer compounds and after half an hour when they were well mixed together, each of the samples was mixed with a specific amount of salt (particle size of NaCl was about more than 100 micron). Then pour it into the mold and let the samples dry completely. After removing them from the mold, we put them in distilled water for a week to remove the salt completely.

### Evaluation of scaffold properties

#### Scanning electron microscopy (SEM)

Morphology, pore size, and nanoparticles distribution of PGS/PCL, PGS/PCL/Gel, PGS/PCL/Gel/HA (3% wt.), and PGS/PCL/Gel/HA (5% wt.) scaffolds were examined by scanning electron microscopy (TESCAN, MIRA III model). The samples were mounted on aluminum stubs and coated with a thin layer of gold. In addition, the morphology and adhesion of the cells on the scaffolds were identified by SEM.

#### Energy-dispersive X-ray spectroscopy (EDX)

Mapping tools are applied for researching Calcium (Ca), Phosphorus (P), and Oxygen(O) atoms as the main elements in the HA nanoparticles. For this purpose, EDX (TESCAN, MIRA II SAMX detector model) was used as a complementary test to investigate the morphology, elemental distribution, and qualitative microanalysis of the samples.

#### Scaffold swelling

The swelling behavior of five composite scaffolds was determined by soaking them in PBS (pH 7.4) solution at 37 °C. The initial weight of the samples was measured and noted as W_0_, then the scaffolds were placed in PBS and the weight of swollen scaffolds (Ws) was measured after the removal of extra PBS by filter paper. The percentage of PBS absorption of the scaffolds was calculated according to the following equation after 24 h [34]:

1 $$Swelling\;rate\;(\%)=\frac{{W}_{s}-{W}_{0}}{{W}_{0}} \times 100$$

#### Contact angle

The water contact angle investigates the surface hydrophilicity behavior of the prepared scaffold. The contact angle was measured using a contact angle measuring system (CAG-20 SE, JIKAN) using four microliter droplets of deionized water on the scaffolds. The angle between the water droplet and scaffold surface was measured after 20 s at room temperature (Image J software) and reported as the contact angle.

#### Scaffold degradation

The scaffold degradation rate is a critical factor in tissue engineering that should be coordinated with tissue regeneration [35]. Hydrolytic degradation of samples was assessed by measuring the initial weights of a specific size (10 mm × 10 mm × 15 mm) of polymeric scaffolds (W_0_) followed by immersing them in 20 ml of PBS solution in a shaker incubator at 37 °C (Heidolph) for desired time intervals (1, 3, 7, 14,28 and 40 days). After each period, the liquid was removed and scaffolds were washed with deionized water 3 times for 15 min and dried in a vacuum oven. Then, the weights of scaffolds were measured (W_t_), and their degradability was calculated according to weight loss by the following formula [36]:

2 $$Degradation(\%)=\frac{{W}_{0}-{W}_{t}}{{W}_{0}} \times 100$$

#### Mechanical properties of scaffolds

To measure mechanical features (compressive test), rectangular sections of scaffold samples with a thickness of 9 mm were cut by scalpel blade at size11 × 9 mm^2^. The compressive test was carried out by a mechanical device (SANTAM, STM-20, Korea) at speed of 2 mm/min at ambient temperature.

### Verification of cellular properties

#### Cell culture and seeding

Human adipose-derived mesenchymal stem cells (h ADSCs) were supplied from the Iranian Biological Resource Center and cultured in a Dulbecco's Modified Eagle's Medium (DMEM; BIOSERA, USA)/F12 containing 10% fetal bovine serum (FBS; Gibco, USA), 100 U/mL penicillin and 100 µg/ml streptomycin (Invitrogen) at 37 °C and 5% CO_2_ in 95% humidity.hADSCs were cultured until reaching a confluence between 70 and 80%. The culture medium was changed every 3 days. Four groups of scaffolds including PGS, PGS/PCL/Gel, PGS/PCL/Gel/HA3%, and PGS/PCL/Gel/HA5% in three replicates were placed in 1 cm × 1 cm wells of 12-well plates in DMEM F-12 medium and fourth passage culture of cells (5 × 10^5^ cells/cm^2^) was seeded on the scaffolds and cultured in the proliferation culture medium for desired times (1,7 and 14 days). The culture medium was changed every 3 days.

Before seeding the cells on the scaffold, a small amount of medium was poured into each well containing the scaffold and placed in an incubator (BINDER, Germany) overnight so that the cells can better adhere to the scaffolds.

#### Cell attachment

The morphology and adhesion of the ADSCs on PGS, PGS/PCL, PGS/PCL/Gel, PGS/PCL/Gel/HA3%, and PGS/PCL/Gel/HA5% scaffolds were identified by SEM (TESCAN, MIRA III model) on day 3 after cell seeding.

#### Cell viability test (MTT)

The cytotoxicity of the scaffolds was evaluated using an MTT assay (3-[4, 5-dimethlythiazol-2- yl]-2, 5-diphenyl tetrazolium bromide). After 1,7 and 14 days of seeding cells on scaffolds, 10 mg MTT was dissolved in 2 ml sterile PBS at a concentration of 5 mg/ml, added to the wells (90 µl medium /10 µl MTT solution), and incubated for 4 h at 37 °C and 5% CO_2_ in 95% humidity. The formazan complex formed during the incubation time was dissolved using 100 µl DMSO (dimethyl sulphoxide). PGS scaffolds were used as controls. The absorption of the obtained solutions was monitored by measuring at 570 nm with an ELISA reader. In this study, the MTT assay was performed on days 1, 7, and 14 of cell seeding, with 3 replicates for each day.

#### RNA isolation and real-time PCR

After in vitro cultivation for 21 days, total RNA was extracted from the cell-scaffold constructs using a standard TRIzol procedure (Kiazist, Iran), according to the manufacturer’s protocol, and the concentration and purity of the RNA were determined using a NanoDrop ND-2000 spectrophotometer (Thermo Fisher, USA). The mRNA was reverse-transcribed into cDNA using an Easy cDNA Synthesis Kit (Parstous, Iran). The expression levels of genes encoding collagen II, aggrecan, Sox9, and Osteocalcin were quantified using RT-PCR on a Real-time PCR thermocycler (ABI Stepone, USA). The sequence of primers (Sinaclon, Iran) that we used in real-time PCR was as Table 2.

**Table 2:** Primer sequences for RT-PCR

Primer Name	Forward sequence	Reverse sequence
**AGGRECAN**	CCTCACCATCCCCTGCTAT	GGGTAGTTGGGCAGTGAGACC
**Col II**	ACCAGGACCAAAGGGACAGAA	GGGCACCTTTTTCACCTTTGT
**SOX9**	AGAAGGAGAGCGAGGAGGACA	CTTGACGTGCGGCTTGTTCTT
**Osteocalcin**	GAAGCCCAGCGGTGCA	CACTACCTCGCTGCCCTCC
**GAPDH**	CTTTGGTATCGTGGAAGGAC	GCAGGGATGATGTTCTGG

The mRNA levels of genes encoding collagen II, aggrecan, and Sox 9 were all normalized to the value of glyceraldehyde-3-phosphate dehydrogenase (*GAPDH*) as an internal control*.* Real-time PCR was performed in one step using SYBR Green Master Mix (SYBR Master with high Rox (2X), Addbio, Korea) under the following conditions: an initial denaturation (95 °C for 5 min) followed by 40 cycles of denaturation (95 °C for 15 s), annealing (60 °C for 15–20 s), and extension (72 °C for 30 s). To verify the reliability of the results obtained, each sample was analyzed in triplicate.

## Results

### Morphological characterization and elemental distribution of scaffolds

**Fig. 2:**
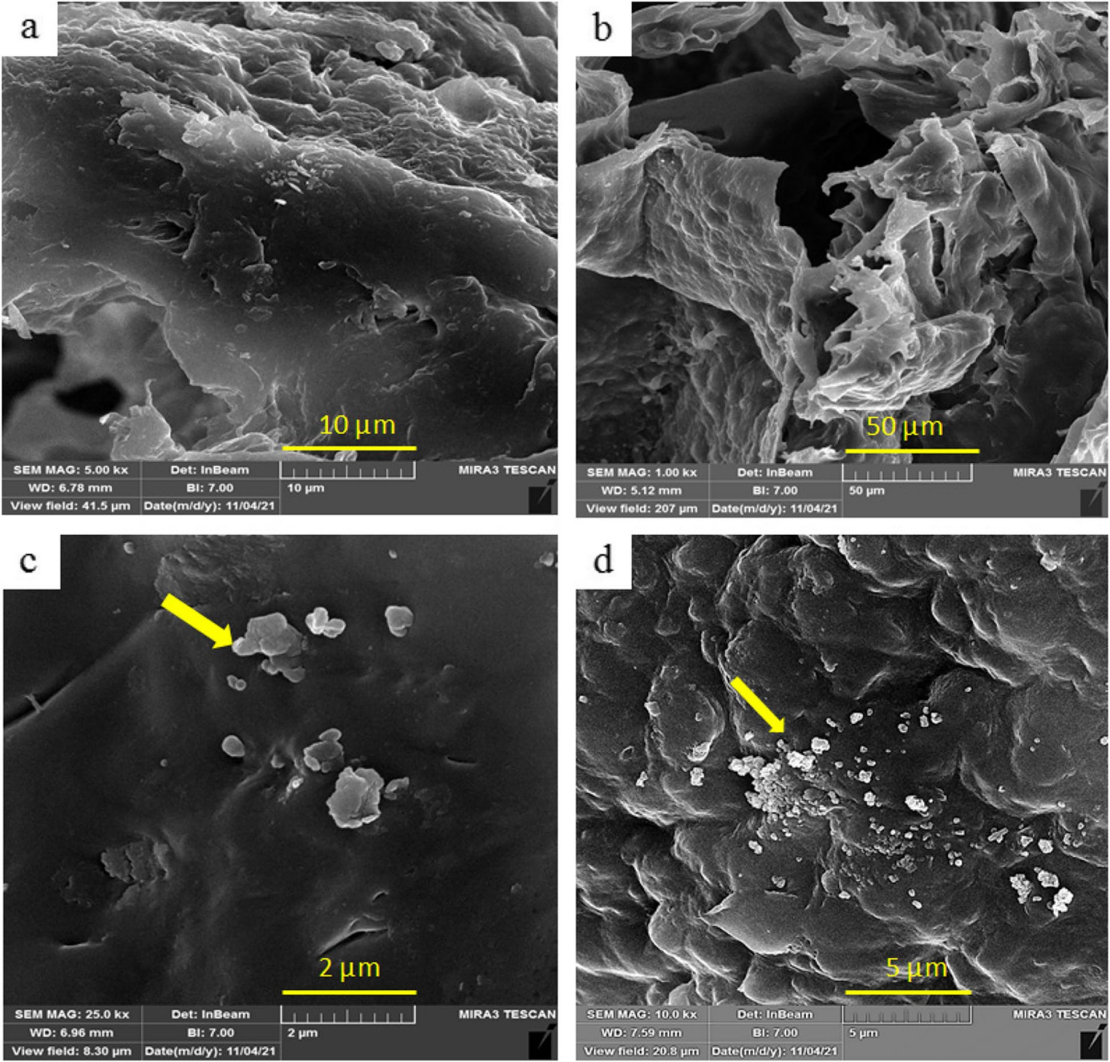
Scanning electron microscopy images of scaffolds fabricated by salt leaching method in the surface, at 5kx magnification. **a** PGS/PCL **b** PGS/PCL/Gel **c** PGS/PCL/Gel/HA 3% **d** PGS/PCL/Gel/HA 5%. SEM images show porous scaffolds with irregular shapes, and HA nanoparticles

Since the morphological characteristics of the scaffolds affect their cellular, mechanical, and thermal behaviors, SEM images from surfaces of PGS/PCL, PGS/PCL/Gel, and PGS/PCL/Gel/HA with different amounts of nanoparticle (3% and 5%) were investigated and shown in Fig. 2. The results showed porous scaffolds with interconnected pores and irregular shapes. As shown in Fig. 2 HA nanoparticles show a monotonous dispersion and excellent adhesion inside the polymeric matrix, indicating a suitable synthesis method.

**Fig. 3:**
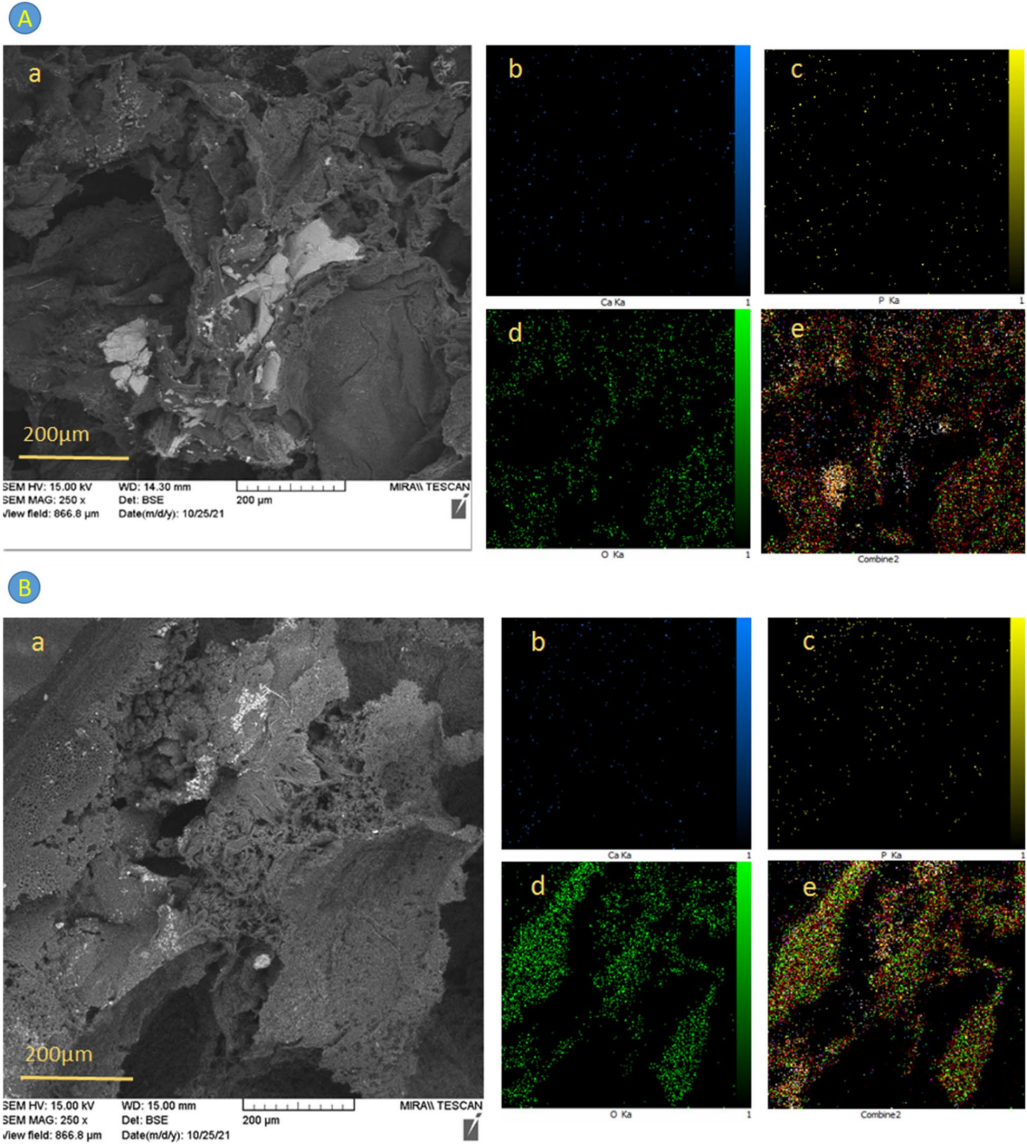
SEM/EDX-Mapping of **A**) PGS/PCL/Gel/HA 3% sample and **B**) PGS/PCL/Gel/HA 5% sample a SEM image b calcium (Ca) atoms c phosphorus (P) atoms d oxygen (O) atoms and e The combination of elements

In addition, the mapping analysis shows the dispersion of calcium (Ca), phosphorus (P), and oxygen (O) atoms as the main elements in HA nanoparticles, so that the atomic percentage of Ca, P, and O in the samples increased with the increase in the percentage of HA nanoparticles. Figure 3A and B represent the dispersion of these atoms in PGS/PCL/Gel/HA 3% and 5%, respectively.

### Hydrophilicity behavior of scaffolds

**Fig. 4:**
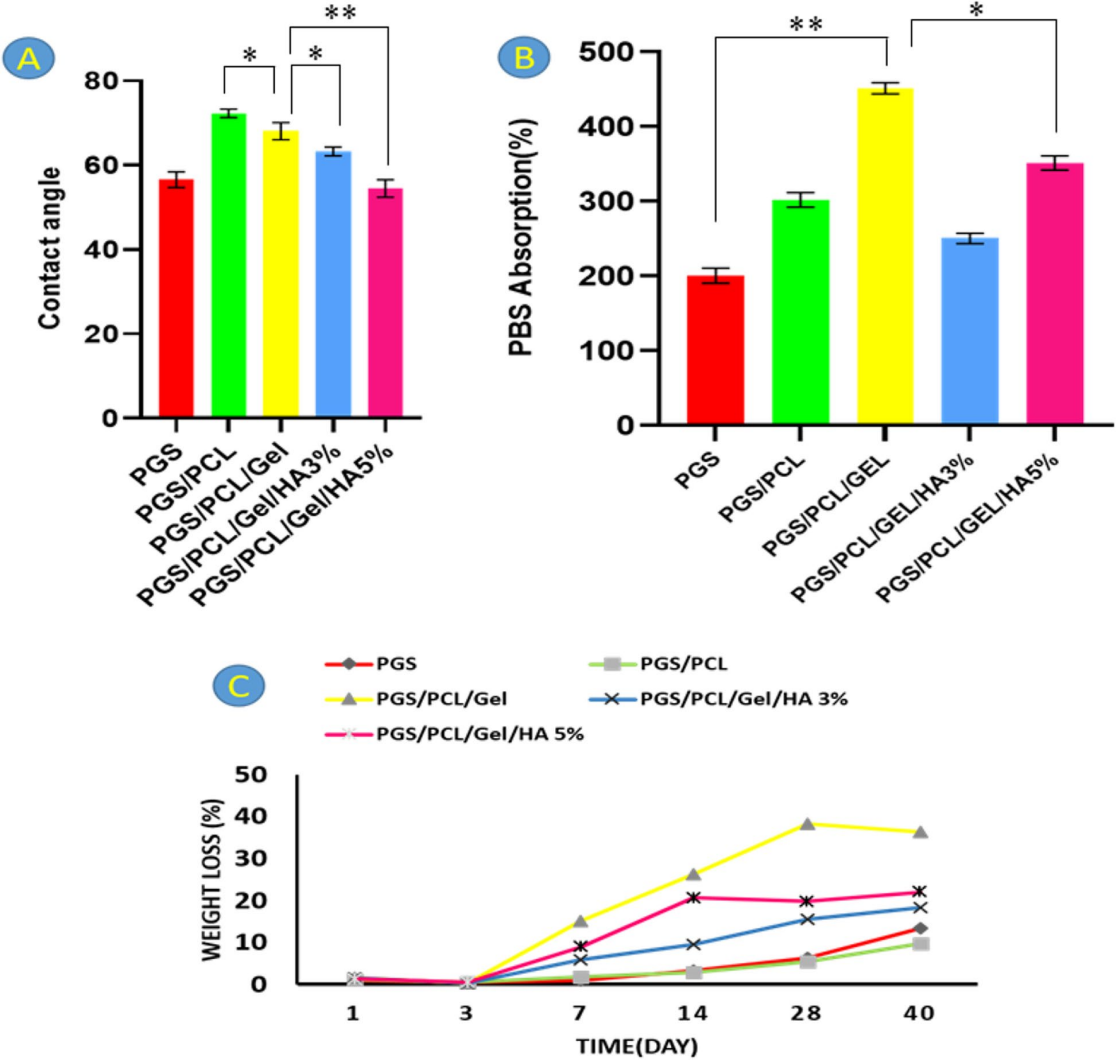
**A** Results from contact angle analysis, **B** The absorption of scaffolds in PBS after 24 h, **C** Hydrolytic degradation of scaffolds in PBS at time interval of 1, 3, 7, 14, 28 and 40 days. The statistical significant analysis paired *t*-test compared to control; *p < 0.05, and **p < 0.01

The hydrophilicity of five composite scaffolds, as an important factor in biocompatibility and biodegradability, was investigated using contact angle analysis [37–39]. The average contact angles for each sample after 20 s have been presented in Fig. 4A. According to the results, the PGS and PGS/PCL/Gel/HA 5% scaffolds as the most hydrophilic samples show a lower contact angle among others. PCL with hydrophobic nature due to aliphatic groups causes an increase in contact angles [37], while gelatin is a natural hydrophilic polymer consisting of a large number of glycine, proline, and 4-hydroxy proline residues, which provides a favorable physico-chemical microenvironment for cell adhesion and proliferation [40].

As shown in Fig. 4A, the contact angle of the PGS/PCL/Gel sample is less than PGS/PCL sample, due to the hydrophilicity of gelatin.

On the other hand, the incorporation of hydroxyapatite nanoparticles with polar groups [41, 42], to the polymer mixture reduces the contact angle compared to the PGS/PCL/Gel scaffold. Among two nanocomposite scaffolds, the incorporation of 5 wt% of HA nanoparticle shows a more significant effect in increasing the hydrophilicity of PGS/PCL/Gel copolymers, so that the contact angle reaches a value lower than the contact angle of the PGS scaffold. These results showed the effect of gelatin and HA nanoparticles on enhancing the hydrophilic properties of composite scaffolds.

The swelling process is defined as an increase in the volume of a gel or solid material due to liquid absorbance**.** In polymers, swelling is the first step of interaction between liquid molecules and the polymeric network, which is usually followed by solving the polymer chains [37]. The swelling behavior of the scaffold is a crucial parameter that can influence cell adhesion and growth [43].

As shown in Fig. 4B, after 24 h, the PBS absorption of all composite scaffolds has significantly increased compared to the PGS scaffold as control. Results revealed that the PGS/PCL/Gel sample has the highest swelling percentage among others.

### Hydrolytic degradation of scaffolds

The degradation behavior of polymeric scaffolds after tissue restoration is a critical feature that eliminates the need for secondary surgeries [43–45]. Since PGS has too fast degradation rate, PCL as a hydrophobic polymer with a low degradation rate, is a good candidate to control its degradation rate and mechanical stability in the body [46–49].

Figure 4C demonstrates the results of degradation during 40 days. Moreover, the combination of PCL with hydrophilic materials including Gelatin and HA nanoparticles increases the degradation rate [43]. Gelatin as a hydrophilic natural polymer enhances wettability, accelerates degradation, and improves cell recognition sites of PCL [12].

In this study, the degradation of pure PGS, PGS/PCL, PGS/PCL/Gel, and PGS/PCL/Gel/HA scaffolds with different contents of nanoparticles (3, 5 wt %) was investigated. As shown in Fig. 4C, PGS/PCL/Gel composite scaffolds show the highest weight losses among others because of their swelling behavior. As expected, scaffolds with higher HA nanoparticle content (5 wt%) showed a higher degradation rate compared to PGS/PCL/Gel/HA 3%, due to the hydrophilic nature of HA nanoparticles.

### Mechanical properties of scaffolds

Cartilage is a composite load-bearing tissue found in animal and human joints [50]. Since ECM regeneration depends on the mechanical properties of the scaffold at both macroscopic and microscopic scales, evaluation of the mechanical properties is a critical factor for providing a stable structure and clinical application of the scaffold [43].

**Fig. 5:**
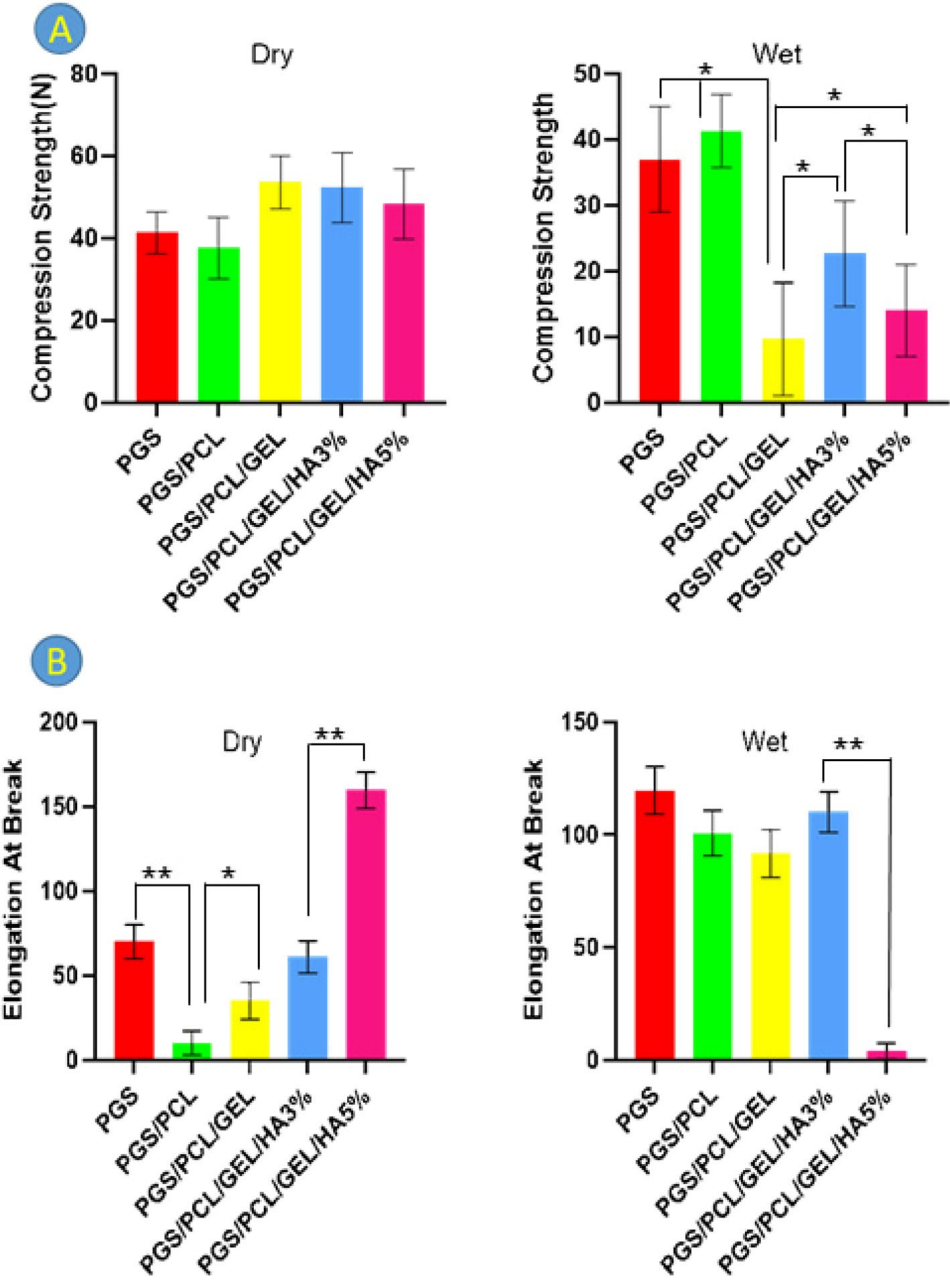
Mechanical properties **A** Compression strength, **B** Elongation at break of the scaffold; at dry (left) and wet (right) conditions; The values are expressed as means (± SEM; *n* = 3), (*p < 0.05), (** *p* < 0.01)

The mechanical characteristics including compression strength (Fig. 5A), elongation at break (Fig. 5B), and module (Table 3) of composite scaffolds were investigated to assess the effect of the combination of PCL and Gelatin as well as HA nanoparticles in PGS-based composites (Fig. 5). Considering dry scaffolds, the results revealed that compression strength of PGS/PCL/Gel and PGS/PCL/Gel HA3 wt % composites are higher than other scaffolds. On the contrary, the compression strength of PGS/PCL/Gel/HA 5% samples in the wet state shows a remarkable decrease (*p-value* ≤ 0.05) compared to PGS/PCL/Gel/HA 3% scaffolds, which can be attributed to the higher swelling in the scaffolds with 5% HA.

**Table 3:** Calculated modulus of fabricated scaffolds

Samples	Modulus (MPa) (Dry)	Modulus (MPa) (Wet)
PGS	0.69	0.39
PGS/PCL	0.71	0.65
PGS/PCL/Gel	1.13	0.16
PGS-PCL-Gel-HA 3%	1.13	0.45
PGS-PCL-Gel-HA 5%	1.30	0.43

No significant changes (*p-value* > 0.05) were observed in compression strength between PGS, PGS/PCL, and PGS/PCL/Gel samples in dry condition, however as shown in Fig. 5A in wet state, the compression strength of PGS/PCL/Gel scaffolds showed a significant decrease (*p-value* ≤ 0.05) compared with PGS, and PGS/PCL samples. Additionally, there is considerable difference (*p* ≤ 0.05) between PGS/PCL/Gel, PGS/PCL/Gel/HA 3%, and PGS/PCL/Gel/HA 5% samples, in the wet state.

As shown in Fig. 5B, the addition of PCL to PGS is associated with a significant decrease (*p-value* ≤ 0.01) in elongation in the dry state, due to the toughness of the PCL, while the addition of gelatin increases elongation (*p-value* ≤ 0.05). Meanwhile, by introducing HA nanoparticles, the elongation represents a significant increase (*p-value* ≤ 0.01), which shows a higher value than the elongation of pure PGS sample by the increase of HA percentage. However, in the wet state, the elongation of the samples in PGS, PGS/PCL, PGS/PCL/Gel, and PGS/PCL/Gel/HA 3%/ scaffolds is not significantly different (*p-value* > 0.05), while with the increase of HA nanoparticles to 5%, a sharp decrease in elongation is observed (*p-value* ≤ 0.01).

### Cell viability and proliferation

**Fig. 6:**
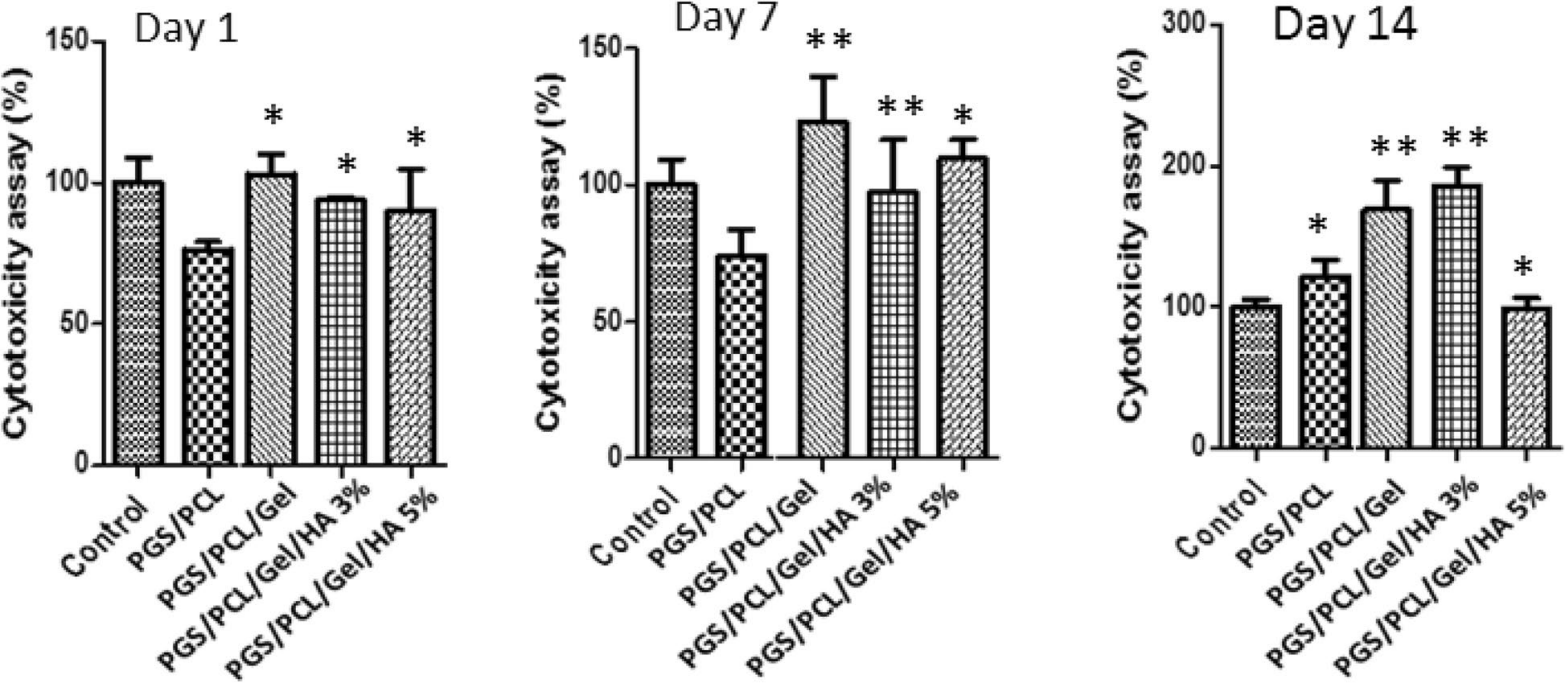
In vitro evaluation of human mesenchymal stem cells survival and proliferation on 1, 3, and 14 days after cell seeding. The statistically significant analysis paired *t*-test compared to control cells. *p < 0.05, and **p < 0.01

To determine the effect of nanocomposite scaffolds on cytocompatibility and cell proliferation, the optical absorbance of ADSCs at 570 nm has been evaluated at the time intervals of 1, 7, and 14 days. According to MTT results, shown in Fig. 6, after 24 h, the engineered scaffolds in PGS/PCL group had inhibitory effects on cell proliferation. After that, on day 7, a significant increase in cell proliferation was observed in the PGS/PCL/Gel and PGS/PCL/Gel/HA 5% groups compared to the control samples (PGS scaffolds). Finally, after 14 days of treatment, all groups except PGS/PCL/Gel/HA 5% showed higher cell proliferation, especially in the presence of 3% HA. Based on these results, PGS-PCL-Gel and PGS-PCL-Gel-HA 3% scaffolds can support more cell survival and proliferation in vitro over time.

### Cell attachment

**Fig. 7:**
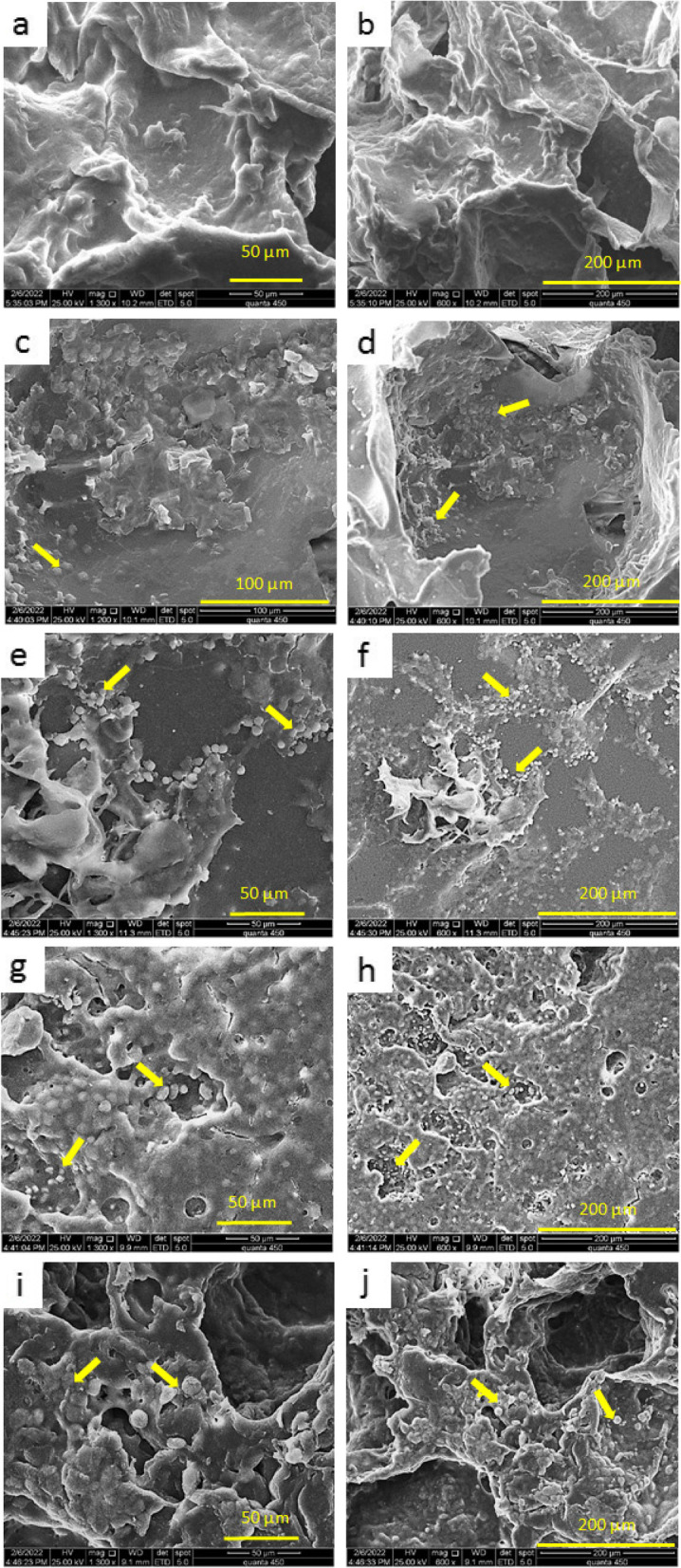
Scanning electron microscopy images of human adipose derived mesenchymal stem cells seeded on the scaffolds 3 days after cell seeding (a&b) PGS, (c&d) PGS/PCL, (e&f) PGS/PCL/Gel, (g&h) PGS/PCL/Gel/HA 3%, (i&j) PGS/PCL/Gel/HA 5%

The cell attachment and behavior of PGS, PGS/PCL, PGS/PCL/Gel, and PGS/PCL/Gel/HA (3 and 5%) scaffolds three days after cell seeding have shown in Fig. 7, revealing the interaction of cells with scaffolds as well as cell attachment and spreading of them inside the pores. Since hydrophilicity and functional groups at the scaffold surface are the major factors in cell adhesion [51, 52], more cell-scaffold interaction can be observed at PGS/PCL/Gel/HA 3%( g, h) and PGS/PCL/Gel/HA 5%,( i, j) due to hydroxyapatite nanoparticles with polar groups. In addition, PGS/PCL/Gel scaffolds (e, f) have a very similar trend in this property, due to the hydrophilicity of Gelatin. These findings are confirmed by contact angle analysis in "Hydrophilicity behavior of scaffolds" section.

### Cell differentiation

**Fig. 8:**
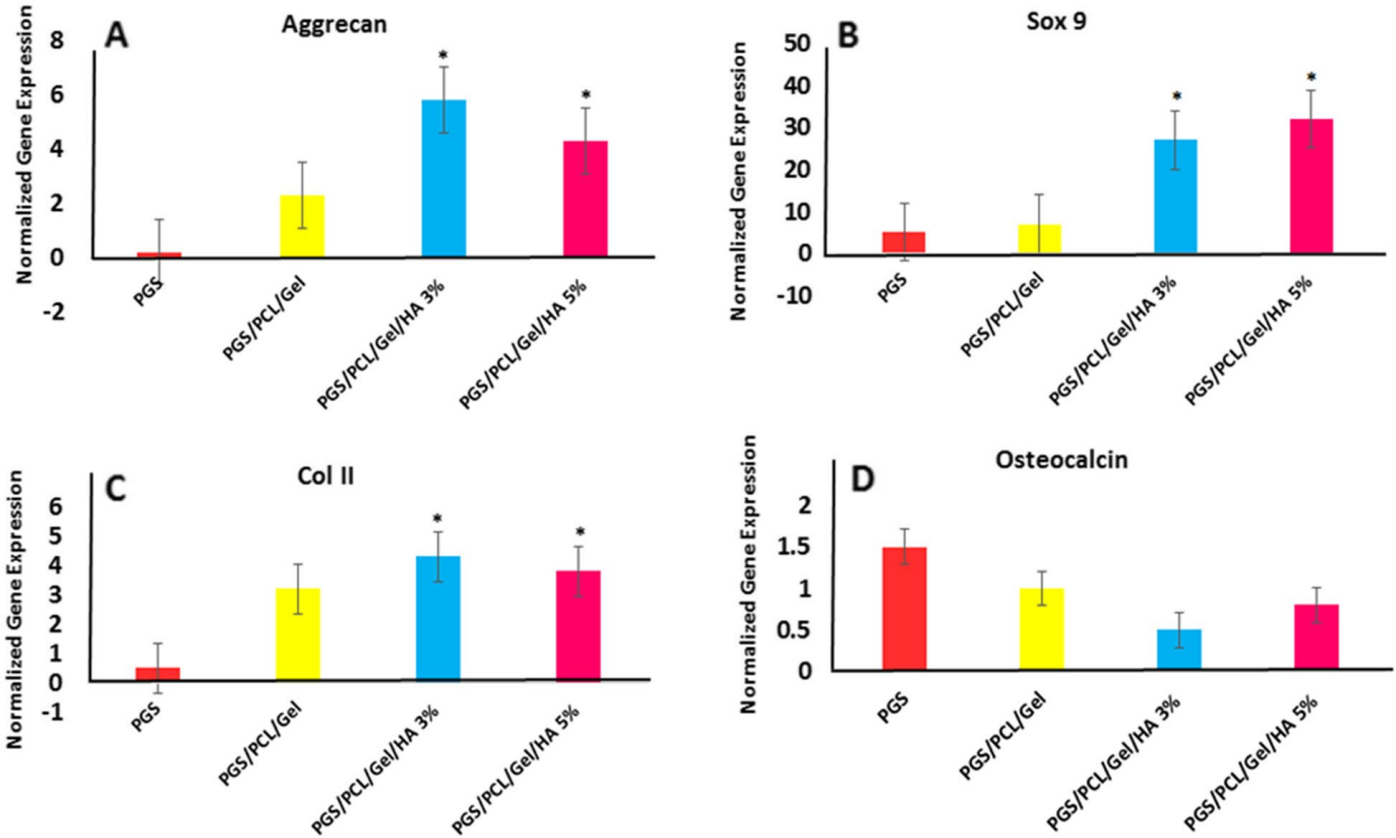
Gene expression profile of chondrogenic markers **A** Aggrecan, **B** Sox9, and **C** Col2 **D** Osteocalcin on PGS and PGS/PCL/Gel, PGS/PCL/Gel/HA 3 & 5%; the results are averages of three independent experiments,* *p*-value ≤ 0.05

To evaluate the chondroconductive capability of the scaffolds, mRNA levels of Aggrecan, Sox9, Collagen II,as well as Osteocalcin were investigated after 21 days of cell seeding and results are depicted in Fig. 8.

The increase of Aggrecan expression as a marker of chondrogenic differentiation was observed after 21 days of seeding for scaffolds with 3 and 5 wt% of HA nanoparticles as compared to PGS and PGS/PCL/Gel samples (*P* < 0.05) (Fig. 8A).

The results showed an increase in the Sox9 gene expression level for samples with 3 and 5 wt% of HA nanoparticles as compared to PGS and PGS/PCL/Gel scaffolds revealing mineralization effect of nHA (*P* < 0.05) (Fig. 8B). As a result, mRNA analysis revealed significant cartilage-related gene expression for samples containing HA, which was consistent with other studies [18–20, 53], indicating the chondroconductive properties of HA. In addition, normalized data in Fig. 8C show the highest increase in Col2 expression in PGS/PCL/Gel/HA 3% and 5% scaffolds compared to other groups (*P* < 0.05), indicating the role of nHA in chondrogenic differentiation. The mRNA expression of Sox9 was also in accordance with the results observed for Aggrecan and Col2 genes. As shown in Fig. 8D,the result of gene expression for Osteocalcin as a bone-related gene showed down-regulation (*P* > 0.05).

## Discussion

Poly (glycerol sebacate) (PGS), due to good mechanical properties, high plasticity, easy processing, and good biocompatibility [54] has been used as tissue engineering scaffolds for many applications [55, 56]. In particular, PGS has shown promising results in nerve, cartilage, skin, bone, and cardiac tissue engineering. However, the low mechanical strength, almost poor hydrophilicity, and fast degradation of PGS via a surface erosion mechanism need to investigate methods to improve its properties. To overcome these issues, blending PGS with other polymers or fillers can result in a material with significant mechanical stability, hydrophilicity, and desired degradability rate [57–59].

For example, creating copolymers with PCL can result in a material with both the strength and elasticity of PCL, while changing the degradation time slightly to fit the scaffold needs [5]. Rostamian et al. indicated that PGS-co-PCL scaffolds possess promising applications for soft tissue engineering, due to adjusting the general features of PGS [37]. In another study by Gaharwar et al., nanocomposites of PGS and CNTs demonstrated that covalent crosslinks between CNTs and PGS considerably enhanced the mechanical properties of PGS [60]. Aghajan et al. prepared a PGS-based nanocomposite scaffold of gelatin, graphene oxide, and clay nanoparticles through in situ polymerization [45]. Furthermore, in a study by Chen et al., the incorporation of halloysite nanotubes into PGS could modify the physical properties of PGS and provide proper conformation and stable mechanical behavior, as well as reduce its degradation rate [61]. Zhao et al. reported that the addition of silica glass particles (SC) into PGS enhances the mechanical properties and hydrophilicity of the PGS-SC hybrid elastomers compared with PGS [62].

Also, Lau et al. showed that hydrophilicity and degradation rate, as well as cell viability, were improved significantly by incorporating β-tricalcium phosphate (β TCP) within PGS [63]. Our results in confirmation of these studies show that the addition of PCL and HA nanoparticles is associated with an increase in mechanical properties, adjustment of hydrophilicity and degradation rate of scaffolds. A large body of evidence has shown the combination of synthetic and natural polymers as well as the addition of hydroxyapatite nanoparticles to improve the differentiation of stem cells into chondrocytes. For example, gelatin as the hydrolysate of collagen, possesses excellent biocompatibility, low immunogenicity, and appropriate biodegradability. It can provide interactions with stem cells through the collagen-binding proteins, and thereby promotes cell proliferation, adhesion, and migration [17, 64–67].

Regarding to PCL, in a study by Wise et al., the oriented electrospun PCL scaffolds induced chondrogenic markers in MSCs and enhanced chondrogenesis [68]. The same results also were observed in a study by Ousema et al., in which 3D woven PCL scaffolds could promote chondrogenesis while maintaining favorable mechanical characteristics, without eliciting pro-inflammatory cytokine [69]. In addition, poly (vinyl alcohol)/polycaprolactone (PVA/PCL) nanofiber scaffolds revealed BM-MSC chondrogenic differentiation and proliferation in vitro and in vivo [70].

Hong et al., developed 3D-printed HA-doped, enzyme-crosslinked gelatin scaffolds, and demonstrated the capability of the scaffolds to support the proliferation and chondrogenic differentiation of human umbilical cord blood-derived mesenchymal stem cells (hUCB-MSCs). Jiang and co-workers also proved that a gelatin/HA film can support growth and preserves the phenotype of chondrocytes compared with gelatin alone [17]. Jamal et al. also showed the promotion of the proliferation and migration of chondrocytes in HA-based colloidal gels [18]. Hybrid materials containing HA also showed chondrogenic differentiation of stem cells. For example, Spadaccio and co-workers developed poly-L-lactic acid/HA electrospun nano-composites which induced chondrogenic differentiation of human MSCs [19]. In a recent report, Calabrese et al. developed gelatin/HAP hybrid materials and demonstrated the chondrogenic differentiation of stem cells [20]. This pattern of enhanced differentiation into cartilage with increased expression of collagen II, aggrecan, and Sox9 genes, and decreased expression of Osteocalcin is also observed in our results, which demonstrates the role of composite compounds of natural and synthetic polymers and HA nanoparticles in the differentiation of stem cells into chondrocytes.

In this study, composite porous scaffolds were fabricated with PGS, PCL and Gelatin with different ratios of hydroxyapatite nanoparticles (3% wt. and 5% wt. nHA) by salting-out method. The results showed that PGS/PCL/Gel/HA scaffolds had great potential for cartilage regeneration. Since, porous structure is essential for the transportation of nutrient/gas and consecutively for cell response [17], our polymeric composites could be fabricated into 3D porous scaffolds with appropriate porosity according to SEM images.

The degradation data demonstrate that the degradation rate of PGS/PCL scaffolds was significantly slower than that of PGS scaffolds. This degradation rate is consistent with the regeneration of the extracellular matrix (ECM) and restoration of mechanical integrity. Thus, the combination of these polymers fulfills the initial requirements for engineering scaffolds in cartilage regeneration.

Based on the mechanical characterization of the scaffolds, the addition of PCL to PGS is associated with a considerable decrease in the scaffolds' elongation in the dry state, which is due to the toughness of the PCL. However, the addition of gelatin increases elongation. Meanwhile, by introducing HA nanoparticles, the elongation represents a significant increase, and this rise reaches a higher value than the elongation of pure PGS scaffolds by the increase of HA percentage. However, in the wet state, the elongation of the samples in PGS, PGS/PCL, PGS/PCL/Gel, and PGS/PCL/Gel/HA 3%/ scaffolds is not significantly different, while with the increase of HA nanoparticles to 5%, a sharp decrease in elongation is observed. Regarding compressive strength in dry state, it can be seen that the addition of gelatin and HA increases it compared to PGS and PGS/PCL samples. While this trend is reversed in wet mode.

As can be seen, Gelatin as a biocompatible natural polymer can improve MSCs attachment on the scaffolds due to interaction with stem cells through the collagen binding proteins. According to the results, the PGS/PCL/Gel and PGS/PCL/Gel/HA 3% scaffolds could support more cell survival and proliferation after 14 days which is confirmed by other studies [60, 70].

Despite, several studies have shown the role of Osteocalcin (OC) in cartilage and bone development by the differentiation of stem cells into chondrocytes to form a cartilage matrix as a scaffold for mineralization, OC is a marker of mature osteoblasts that is not found in resting zone or adult cartilage. Therefore, in this study, we investigated the expression of OC by ADSCs, which confirms cartilage differentiation instead of bone differentiation [71].

According to previous studies, SOX9, COLII, Aggrecan, and COMP as cartilage-related genes were up-regulated in hMSCs undergoing chondrogenic differentiation, while osteogenic genes (COLI, Osteocalcin) were down-regulated [72].

The expression of collagen type II and aggrecan as specific and main components of cartilage matrix are regulated by three Sox transcription factors, including Sox5, Sox6 and Sox9 [73].

According to our results, there is an remarkable increase in mRNA levels of collagen II, aggrecan, and Sox9 as markers of chondrogenic differentiation, and a significant decrease in Osteocalcin expression, which is consistent with previous studies [17].

## Conclusion

In the present study, PGS/PCL/Gel scaffolds as new biomaterials have been synthesized with and without hydroxyapatite nanoparticles to compare with pure PGS and PGS/PCL samples. The morphology of all nanocomposites was evaluated by SEM and EDX images, demonstrating proper dispersion of nanoparticles within polymeric scaffolds. As shown by contact angle measurement, the hydrophilic behavior was influenced by incorporating Ɛ-caprolactone which causes a decrease, and Gelatin and HA nanoparticles cause an increase in the hydrophilicity of the copolymers. Furthermore, both PGS/PCL/Gel samples with 3 and 5% HA possess appropriate biocompatibility and enhanced cell proliferation and attachment. Our results also proved the differentiation of hADSCs according to mRNA expression levels of collagen II, aggrecan, and Sox9. It can be concluded that PGS-based nanocomposites, including 3 and 5% HA, are considered an appropriate scaffold for cartilage regeneration applications.

## Data Availability

The raw/processed data required to reproduce these findings are available on request from the corresponding author. The data are not publicly available at this time as the data also forms part of an ongoing study.
