# Correction: The role of three‑dimensional scaffolds based on polyglycerol sebacate/ polycaprolactone/ gelatin in the presence of Nanohydroxyapatite in promoting chondrogenic differentiation of human adipose‑derived mesenchymal stem cells

**DOI:** 10.1186/s12575-023-00209-y

**Published:** 2023-05-17

**Authors:** Pardis Yousefi Talouki, Saeed Hesami Tackallou, Shahrokh Shojaei, Soheila Zamanlui Benisi, Vahabodin Goodarzi

**Affiliations:** 1 Department of Biomedical Engineering, Central Tehran Branch, Islamic Azad University, Tehran, Iran; 2 Department of Biology, Central Tehran Branch, Islamic Azad University, P.O. Box 13145‑784, Tehran, Iran; 3 Stem Cell Research Center, Tissue Engineering and Regenerative Medicine Institute, Islamic Azad University, Central Tehran Branch, Tehran, Iran; 4 Applied Biotechnology Research Center, Baqiyatallah University of Medical Sciences, Tehran, Iran

**Correction: Biol Proced Online 25:9, (2023)**

**https://doi.org/10.1186/s12575-023-00197-z**

Following publication of the original article [1], the authors identified mistakes on affiliations 1, 2 and 3, Central Branch should be corrected to Central Tehran Branch.

The corrected affiliations are shown in this article and the original article has been corrected.
